# Prognosis of anomalous coronary arteries originating from the opposite sinus of Valsalva (ACAOS): 15 year experience from two large CMR centres

**DOI:** 10.1186/1532-429X-15-S1-P257

**Published:** 2013-01-30

**Authors:** David P Ripley, Albert Teis, Akhlaque Uddin, Petra Bijsterveld, Ansuman Saha, Ananth Kidambi, Bernhard A Herzog, Sven Plein, Dudley J Pennell, John P Greenwood

**Affiliations:** 1 Multidisciplinary Cardiovascular Research Centre (MCRC) & Leeds Institute of Genetics, Health and Therapeutics, University of Leeds, Leeds, UK; 2 Leeds General Infirmary, Leeds Teaching Hospitals NHS Trust, Leeds, UK; 3 Cardiovascular Magnetic Resonance Unit, Royal Brompton Hospital, London, UK

## Background

Aberrant coronary arteries represent a diverse group of congenital disorders, many of which may have no clinical significance. Post mortem studies show a high risk of exercise related sudden cardiac death in those with an anomalous coronary artery originating from the opposite sinus of Valsalva (ACAOS) that takes an inter-arterial (anterior) course. However, there is little documentation in life of the long term natural history of anomalous coronary arteries.

## Methods

Databases from two cardiovascular magnetic resonance (CMR) centres (Leeds General Infirmary and Royal Brompton Hospital) were reviewed. Patients with anomalous coronary arteries undergoing CMR over a 15 year period (1995 to 2009) were identified. Anomalous coronary arteries were classified according to their anatomy and course. Both the electronic and paper records of all patients were reviewed for major adverse cardiovascular events (MACE) defined as cardiovascular mortality, myocardial infarction, revascularisation (PCI or CABG). Cause of death was verified by the Office for National Statistics. Revascularisation or myocardial infarctions were only counted if they occurred in the distribution of the anomalous artery.

## Results

173 consecutive patients with coronary artery anomalies were retrospectively identified with a median age 54 years (range 1-85). The median follow-up time was 4.3 years (IQR 2.5 - 7.8) with a maximum of 15.6 years. Of the 173 patients, 117 had ACAOS of which 111 were alive, 5 deceased and 1 lost to follow-up. 65 patients (56%) had an inter-arterial course and 52 (44%) a posterior course. The distribution of coronary anomalies is detailed in Table 1.

**Table 1:** Classification of anomalous coronary arteries (n=173)

Classification	Number
**1. Origin of both RCA and LMS (separate origins) from the right aortic sinus**	

- 1a. Course of anomalous LMS between aorta and RVOT (anterior)	13

- 1b. Course of anomalous LMS not between aorta and RVOT (posterior)	8

**2. Origin of both coronary arteries (separate origins) from the left aortic sinus**	

- 2a. Course of anomalous RCA between aorta and RVOT (anterior)	34

- 2b. Course of anomalous RCA not between aorta and RVOT (posterior	0

**3. Anomalous origin of the circumflex coronary artery from the right aortic sinus**	

- 3a. Course of anomalous LCx between aorta and RVOT (anterior)	1

- 3b. Course of anomalous LCx not between aorta and RVOT (posterior)	33

**4. Anomalous origin of the left anterior descending artery from the right aortic sinus**	

- 4a. Course of anomalous LAD between aorta and RVOT (anterior)	4

- 4b. Course of anomalous LAD not between aorta and RVOT (posterior)	6

**5. Single coronary artery (common origin)**	

- 5a. Course of anomalous coronary artery between aorta and RVOT (anterior)	13

- 5b. Course of anomalous coronary artery not between aorta and RVOT (posterior)	5

**6. Anomalous origin or communication of a coronary artery with a cardiac chamber or major thoracic vessel**	

- 6a. Abnormal origin from the pulmonary artery or one of its major arterial branches	11

- 6b. Abnormal origin from the aorta or one of its major arterial branches	2

- 6c. Abnormal communication of a coronary artery with a cardiac chamber or major thoracic vessel (fistula).	18

**7. Miscellaneous / unclassified**	25

In those patients with ACAOS there were 59 MACE events (5 cardiovascular deaths, 6 PCI, 24 CABG and 24 had myocardial infarction). 48 MACE events occurred in ACAOS with an anterior course and 11 with a posterior course (p<0.0001) the statistical difference driven by surgical revascularisation and myocardial infarction (Table 2).

**Table 2:** Major Adverse Cardiovascular Events in patients with an anomalous coronary artery originating from the opposite sinus of Valsalva (ACAOS) (n=117)

	Anterior (n=65)	Posterior (n=52)	P value
Cardiovascular Deaths, n	3	2	NS

PCI, n	4	2	NS

Surgical revascularisation, n	23	1	P<0.0001

Myocardial Infarction, n	18	6	P<0.05

## Conclusions

We have demonstrated that in life, patients with an anomalous coronary artery originating from the opposite sinus of Valsalva taking an anterior course, have higher rates of both myocardial infarction and surgical revascularisation during long-term follow up, compared to those with a posterior course.

## Funding

S.P is funded by a British Heart Foundation fellowship (Fs/10/62/28409) S.P. and J.P.G received an educational research grant from Philips Healthcare.

